# Addressing health inequalities and barriers to access among adolescent migrants in Chile: a mixed methods study

**DOI:** 10.1186/s12939-025-02615-y

**Published:** 2025-09-26

**Authors:** Alejandra Carreño-Calderón, Alexandra Obach, Baltica Cabieses, Marcela Oyarte, Alicia Arias Schreiber

**Affiliations:** 1 Centro de Salud Global Intercultural, Facultad de Medicina Clínica Alemana, Facultad de Psicología, Universidad del Desarrollo, Santiago, Chile; 2 Instituto de Salud Pública de Chile, Santiago, Chile; 3 Departamento de Salud y Pueblos Indígenas e Interculturalidad, División de Políticas Públicas Saludables y Promoción, Subsecretaría de Salud Pública, Ministerio de Salud, Santiago, Chile

**Keywords:** Migrant health, Adolescent health, Adolescent health services, Pregnancy in adolescence

## Abstract

**Background:**

The presence of adolescents in migration flows through Latin America and Caribbean (LAC) has increased in recent years. Adolescents are usually considered healthy due to their low mortality rates compared to the general population. However, existing research shows that adolescence is a phase of life in which mental health, sexual and reproductive health and other needs may increase. Migration, as a social determinant of health, can lead to experiencing compounded vulnerabilities among adolescent migrants, especially those already living in disadvantaged conditions.

**Objective:**

aims to estimate and compare social inequalities in health faced by adolescent migrants from LAC living in Chile versus locals, as well as to unveil perceptions and experiences related to additional barriers to accessing to healthcare in the country.

**Method:**

A mixed methods study was designed to socially and epidemiologically characterise the adolescent migrant population in Chile. First, two population-based surveys (CENSO 2017; CASEN 2022; REM 2021 and EH 2021) and national epidemiological records were analysed. Second, perceptions and experiences of accessing primary healthcare services were explored through 42 in-depth interviews with healthcare teams (*n* = 18) and parents of adolescent migrants (*n* = 24). Quantitative and qualitative data were analysed separately and then integrated to identify the main findings. The study was approved by the Ethics Committee of the Universidad del Desarrollo.

**Results:**

The study identified social inequalities negatively affecting adolescent migrant compared with their Chilean peers, including lower access to education, housing and higher chances of having to work. Regarding health, sexual and reproductive needs and experiences are identified, including adolescent pregnancy. Barriers to access to primary healthcare programmes dedicated to adolescent health, which are little known and underused by the migrant population, were also identified.

**Conclusions:**

Adolescent migrants in Chile face important social inequalities in health compared to locals and additional barriers to exercise their right to health, putting their current and future health at risk. Priority actions are needed for this specific group, and must focus on increasing the acceptability and coverage of preventive care, as well as strengthening their participation in the social and health decisions affecting them.

## Background

International migration has experienced significant growth in recent years in Latin America and the Caribbean (LAC). Countries in the Southern Cone have changed from sending to receiving migrants, in a climate marked by the effects of the COVID-19 crisis and the regional tightening of migration policies. Currently in Chile, 10.3% of the population is an international migrant, with the largest groups coming from Venezuela, Peru, Colombia and Haiti [1]. Within the migrant population, adolescents are often overlooked. Adolescence is the phase of life between childhood and adulthood, and includes ages 10 to 19 [2]. A recent report indicates that one third of migration flows in LAC are made up by adolescents and young people between the ages of 12 and 24, with or without their parents or legal guardians [3]. In Chile, 15.8% of the migrant population is between the ages of 0 and 19, and of these, 67.7% are adolescents (10–19 years) [1].

In terms of healthcare, Chile has a fragmented system, characterized by a public insurance fund (FONASA) and a private one (ISAPRE). Since 2017, a regulation has been in place that guarantees access to public health services for the migrant population, regardless of their migration status. As of 2022, 72% of the general migrant population were users of FONASA, 10% were affiliated with ISAPRE, and 16% had no health coverage of any kind [4]. Regarding adolescents, Chile has a special healthcare program dedicated to adolescents (*Programa de Salud Integral de Adolescentes y Jóvenes*), but it has low coverage among vulnerable groups and doesn’t consider the impact of migration on the health status of international migrants. A recent report from the Ministry of Health states that, as of 2021, 4.4% of adolescents enrolled in the Comprehensive Health Program for Adolescents and Youth were migrants, highlighting ongoing challenges in terms of access to and utilization of these services by the migrant population [5].

The lack of data on adolescent migrants in LAC is partly because adolescence is often seen as a healthy stage with low healthcare needs and mortality rates [5, 6]. However, recent research shows the urgency of prioritising this group due to the importance that nutrition, physical activity, substance abuse prevention, and sexuality acquire during adolescence [6]. In the case of economically and socially marginalised groups, such as some groups of migrants of Latin American and Caribbean origin, these aspects may be affected by migration, globally recognised as a social determinant of health [7]. Scientific literature has tended to focus on the situation of Mexican and Central American adolescents migrating to the United States, Canada and some European countries. Even without being located in the Southern Cone, this research shows that the intersection between migration and adolescence in contexts of vulnerability leads to specific needs regarding of mental health [8], sexual and reproductive health [9], nutritional health, drug and alcohol use [10], in addition to raising barriers to access and acceptability of health services.

In terms of mental health, for example, it has been reported that 75% of mental illnesses, excluding dementia, begin before the age of 18 [11] and that adolescence is a key stage in preventing the development of serious psychiatric pathologies [10]. In the case of adolescent migrants, exposure to violence, low socio-economic levels, food insecurity, stigmatisation and xenophobia may increase the likelihood of facing mental health problems [8, 12]. Regarding sexual and reproductive health, some of the global challenges faced by adolescents include early pregnancy and parenthood [12], as well as difficulties to access contraception and safe abortion, and to prevent HIV and sexually transmitted infections [13]. In addition, it has been recognised that healthcare workers can become a barrier to care by failing to provide young people with non-judgemental support services appropriate to their needs [13–15]. Even if all adolescents may experience these challenges, barriers such as lack of knowledge of the health system, fear of being reported or discriminated against, and exposure to racism, are specific to adolescent migrants, and have not been sufficiently studied in LAC [9, 16]. A recent study in Chile showed that adolescent migrants, specifically those of African descent, report situations of racism, stigma and discrimination when accessing sexual and reproductive health care and services because, based on racial stereotypes, they are expected to have early sexual initiation and promiscuous sexual lives [17]. Regarding teenage pregnancy, factors such as limited socio-economic resources, low levels of schooling and having teenage parents have been found to increase the likelihood of early childbearing [18]. Even if it is not possible to associate the migration factor with teenage pregnancy, a study in Colombia revealed a higher probability of teenage pregnancy in municipalities with higher rates of forced displacement [19], while in the United States, another study found that the counties in the country with the highest teenage birth rates were also rural counties with a high percentage of Latino population [20].

In this context, this article aims to estimate and compare social inequalities in health faced by adolescent migrants from LAC living in Chile versus locals, as well as to unveil perceptions and experiences related to additional barriers to accessing to healthcare in the country, based on a mixed methods study [21]. The main aim of the study was to identify barriers and facilitators of access to, and cultural relevance of primary healthcare among migrant children and adolescents, based on the perception of their families and healthcare teams in Chile. In this article we focus on results regarding the health of adolescent migrants (10–19 years old). The study was carried out in Chile in 2023–2024. The article is relevant to the country and the Latin American region, as it describes vulnerabilities and inequities affecting adolescent migrants, compounded by the context of the health and migration crisis that characterised the pandemic and post-pandemic period.

## Method

### Study design

A mixed methods design was carried out with a quantitative descriptive component and a qualitative exploratory component [21, 22]. The quantitative component uses databases from 2017 to 2021 that were available in 2023. The qualitative component uses data collected in 2023. Both components were analysed simultaneously as they were focusing on specific aspects of the research objective. The quantitative component addressed the social and epidemiological conditions of migrant adolescents in Chile, while the qualitative component examined the relationship between migrant communities and primary healthcare services dedicated to the adolescent population.

### Quantitative component

This component described and compared the social and epidemiological situation of migrant adolescents using data from population-based surveys and epidemiological registry databases made available by the Department of Health Statistics and Information (DEIS), Ministry of Health, Chile (MINSAL). Where possible, the information was disaggregated by age group in order to isolate data for the 10–19 years segment, contrasting migrant adolescents with local adolescents, prioritising socio-demographic and epidemiological data.

### Materials

We analysed the most recent population-based data available in the country at the time the study was conducted: the 2017 census and its projections 2018–2021; the National Socioeconomic Characterisation Survey 2020 (*Encuesta de Caracterización Socioeconómica Nacional* CASEN survey thereafter); the Hospital Discharges Records (*Egresos Hospitalarios*, EH thereafter) for the year 2021; and the Monthly Statistical Records for Public Primary Care (*Registro Estadístico Mensual*, REM thereafter) for the same year. These databases are free to access from the Chilean government upon completing online request forms.

Census: The Population and Housing Census 2017, with reduced objectives and thematic hierarchisation, included everyone present in Chile on the 19^th^ of April 2017. Three types of questionnaires were used, according to the type of dwelling or situation. The information was collected according to political-administrative and census divisions, allowing analysis by region and urban or rural area. Particularly for migration, between 2018 and 2021, the National Statistics Institute (INE) and other institutions prepared estimates of the foreign population living in Chile, disaggregated by sex, age and country of origin, considering habitual residence as equal to, or greater than six months, in a process of continuous methodological revision. For the census, a migrant was defined as anyone who specified a country of birth other than Chile, through the question"When you were born, in which municipality or country did your mother live?”. For 2017, the total population of Chile was estimated at 17,574,003, including 784,685 international migrants.

CASEN survey 2020; The National Socio-economic Characterisation Survey is the main instrument for measuring income poverty in Chile, and has been applied every two or three years since 1987. In 2020, due to health restrictions imposed by the COVID-19 pandemic, the survey was implemented through a mixed and sequential modality that combined face-to-face and telephone phases. The sample design was probabilistic, stratified and clustered, with national, regional and geographical (urban-rural) representativeness. The questionnaire, administered to a household member aged 18 or over, was reduced to seven modules focusing on key variables such as education, employment, income and health, prioritising the measurement of poverty and income. The database is anonymised and excludes people living on the street or in institutions. In 2020, 62,540 households were surveyed, corresponding to 62,911 households and 185,437 individuals (8,857 migrants, 173,462 non-migrants y 3,118 didn’t know), expanded to 19,545,799 individuals (1,191,601 migrants, 17,972,203 non-migrants and 381,995 didn’t know), with a response rate of 63.1%. Migrants are defined as those who answer ‘In another country’ to the question"When you were born, in which municipality or country did your mother live?

Hospital Discharge Records (EH): The EH database compiles information from all secondary health centres in Chile, public and private, considering as discharge the departure of a patient after occupying a hospital bed. The data, collected by the Ministry of Health (MINSAL), include demographic variables, health prognosis, diagnosis (ICD-10), discharge condition, surgical intervention, and days of stay. All data is anonymised. A total of 1,371,145 HDs were recorded for 2021 (44,088 for migrants and 1,327,057 for non-migrants). Those who were registered as foreigners are considered migrants.

REM: The Monthly Statistical Records for Public Primary Care register the activities carried out in public health facilities in Chile, organised in different series (A, BS, BM, D and P) where each series is disaggregated into worksheets corresponding to the type of record they contain and each REM sheet contains several sections. These records are compiled monthly and detail the activities carried out in healthcare centres. REM data is organised into different series, collating Excel files or spreadsheets containing different types of records according to subject area or type of service (Series A: Closed care activities, BS: Oral health, BM: Mental health, D: Diagnostic and therapeutic support activities, P: Population receiving check-ups under different health programmes). Specifically, information was extracted on pregnant women with check-ups for biopsychosocial risks, work and studies among the adolescent population receiving check-ups, areas of risk in the adolescent population (sexual and reproductive health, suicidal ideation, suicide attempts, alcohol and other drug use, nutritional status and other clinically identified risks). For all the cases mentioned, information on migrants was available and specified within the files, either through specific columns or differentiated sections.

### Data analysis

Analysis of the social and epidemiological characteristics of international migrant adolescents was carried out in parallel with local adolescents. With regards to living conditions and socio-demographic and economic characteristics, the following dimensions were considered: (i) demographic (sex, age, ethnicity, rurality, region of residence, marital status) and (ii) socio-economic (equivalent household income, educational level, rate of attendance for primary, secondary and higher education, occupation, housing conditions, overcrowding index). In addition to using CASEN 2020, in order to analyse access to healthcare through the characterisation of epidemiological profiles, hospital discharges (EH) and Monthly Statistical Record (REM) databases for 2021 were used. From the hospital discharge (EH) databases, the EHs corresponding to foreigners (used as a proxy for the migrant population) were described in parallel with those corresponding to nationals, according to demographic characteristics (sex, region of residence and age brackets) and main diagnosis (according to the ICD-10 chapters classification). Such descriptions were carried out for the total population and in greater detail of the main diagnosis for the target population of the study. The results were further stratified by sex, age group, educational level and health provision, according to relevance and availability in the respective databases. From the stratification by age group, it was possible to establish comparisons between local and migrant adolescents through Chi-square and T-test with Rao and Scott correction [22] for sample survey data.

### Qualitative component

This component included an exploratory focus on experiences of access to primary healthcare services, from the perspective of migrant adults from LAC living in Chile and healthcare teams (migrant caregivers of adolescents and healthcare primary professionals who work, among others, with migrant populations). For reasons related to ethical requirements and limited data collection time, it was not possible to involve migrant adolescents in the study. Currently there are ongoing studies especially designed to involve them through participatory research methods. A convenience sample of 42 semi-structured interviews was carried out in three regions of Chile, according to specific inclusion criteria for each group (interview guides available in supplementary file; interview transcripts are available by request) Table 1 displays further detail. The sample size was determined by criteria of information saturation and feasibility, considering that both the migrant population and healthcare teams tend to have little time available to participate in social research. As this is an exploratory qualitative study, the sample does not aim to be representative.

**Table 1:** Dimensions of semi-structured interviews guidelines

Dimension	Migrant adults	Healthcare teams
Experience	Migratory experiences (origin, trajectory and current living conditions)	Experience working on healthcare of migrant people
Healthcare needs	Healthcare needs of themselves and their adolescents	Healthcare needs of migrant people and migrant adolescents
Access and use of healthcare services	Barriers and facilities of access to health care for themselves and their adolescents	Barriers and facilities of access to health care for migrant adolescents
Adolescents program	Knowledge and use of Adolescents program, cultural concepts of adolescence	Experiences of attention of migrant adolescents
Other healthcare issues	Use of sexual and reproductive programs, nutritional programs, mental health program, among others	Use of sexual and reproductive programs, nutritional programs, mental health program, among others

### Materials and participants

A convenience sample consisting of 42 in-depth interviews with healthcare professionals (*n* = 18) and parents and guardians of migrant adolescents (*n* = 24) in three regions of Chile, all of them with high representation of migrant people: Tarapacá Region, Metropolitan Region and O’Higgins Region. Inclusion criteria for the group of adult migrants were (a) adult migrants of Latin American and Caribbean origin, (b) any gender, (c) residing in Chile for less than 10 years, (d) caregivers of at least one adolescent between 10 and 19 years old. The group of healthcare teams included professionals and technicians who: (a) provide direct care to migrant children and adolescents; (b) are part of primary healthcare services; b) have been in their position for at least one year. Due to recruitment practical conditions, which was carried out in collaboration with an NGO focused on migration issues, the sample consisted of adults from Venezuela, Bolivia, Peru, Haiti, Argentina, and Colombia. These are the most prevalent countries of origin of international migrants in the country. Table 2 provides further detail of the qualitative sample.

**Table 2:** Qualitative sample by groups of migrant adults and health teams, characterised sociodemographically

Migrant adults	Tarapacá Region	Metropolitan Region	O’Higgins Region
Total	*n* = 8	*n* = 8	*n* = 8
Gender	8 women	7 women, 1 man	8 women
Average age	30 years old	32 years old	36 years old
Nationality	5 Venezuelan	5 Venezuelan	7 Venezuelan
	2 Bolivian	2 Argentinian	1 Colombian
	1 Peruvian	1 Haitian	
*Healthcare teams*			
**Total**	*n* = 6	*n* = 6	*n* = 6
Gender	4 women, 2 men	6 women	3 women, 3 men
Profession	4 Social worker	3 Social worker	4 Social worker
	1 Nurse	1 Nutricionist	2 Cultural mediator
	1 Psychologist	1 Midwife	1 Nurse
		1 Nurse	

### Recruitment and data collection

Adult migrants were contacted through non-governmental organisations (NGO) and healthcare teams were invited to participate through healthcare service authorities who collaborated with the study. The interviews were carried out using a guide prepared by AC and AO (Table 1). The interviews with healthcare teams were conducted online through Zoom and the interviews with adult migrants were conducted face to face in public spaces, in their homes or workplaces or in spaces offered by the NGOs that collaborated with the research, securing privacy and confidentiality. The interviews were conducted in Spanish by the authors (AC and AO) and lasted between 50 min and two hours. All of them were audio recorded and fully transcribed after securing informed consent from the participants.

### Data analysis

Once the information had been transcribed verbatim and checked against original records, a thematic analysis was carried out, allowing to interpret content based on themes, categories and indicators previously designed by the researchers, using the specific objectives and the guiding questions applied in the research instruments as a basis. The Atlas.ti software was used to support the coding and analysis of the information. The coding was carried out both through pre-established categories based on the research objectives, the main topics of the interview guides, and emerging codes. The whole team then reviewed the codification process, discussed discrepancies and agreed on a final version. All codification decisions were approved by the whole research team. A native English speaker translated the quotations into English, and all authors verified them to ensure their original meaning was preserved. Once the results of the quantitative and qualitative components were obtained through a separate analysis, findings in common topics were integrated during the interpretation phase, in order to provide a more comprehensive understanding of the research problem [23].

### Ethics

The study was approved by the Ethics Committee of the Universidad del Desarrollo (Code #2022-92) and respected the ethical principles of human subject research. All interviews were confidential and data were anonymised in the transcription process. The interviewees were informed of the characteristics and implications of the study and signed an informed consent form expressing the voluntary nature of their participation. The research contemplated the implementation of referral protocols in case of general health needs, mental health, migration counselling or in the event of the identification of situations of violence, abuse of children or adolescents and/or situations of human trafficking or smuggling. None of the interviews required activating the protocol.

## Results

The results are divided into three dimensions (I) social vulnerability and inequities affecting adolescent migrants; (II) epidemiological profile and health needs of adolescent migrants and (III) barriers to access primary healthcare programmes dedicated to adolescent health from the perspective of adult migrants and healthcare teams.

### I) Social vulnerability and inequalities affecting adolescent migrants

The results of the 2017 census found that 15.3% of the migrant population was under 19 years old and 58.4% of them were adolescents (10–19 years old). The census also indicated that, in 2017, the majority of migrant children and adolescents at the national level (95.3%) lived in urban areas and that migrant adolescents had a slightly higher percentage of female population compared to Chilean adolescents (49.02% v/s 48.9%). (Table 3 Census 2017 results by sex, age and geographic area).

**Table 3:** Census 2017 results by sex, age and geographic area

		**International Migrants**				**Non Migrants**			
		0 a 4 years	5 a 9 years	10 a 19 years	Total (0-19)	0 a 4 years	5 a 9 years	10 a 19 years	Total (0-19)
Total	Total country	19373	30628	70307	120308	1094816	1148850	2270746	4514412
	R. Tarapacá	1232	2057	5020	8309	23935	24082	42299	90316
	R. O’Higgins	368	566	1370	2304	58006	63709	121537	243252
	R. Metropolitan	12345	18551	41561	72457	435227	439992	872848	1748067
Sex	Male								
	Total country	9765	15500	35840	61105	557781	586587	1159372	2303740
	R. Tarapacá	612	1054	2557	4223	12304	12309	21705	46318
	R. O’Higgins	193	291	715	1199	29524	32484	62306	124314
	R. Metropolitan	6229	9413	21135	36777	221748	225019	443550	890317
	Female								
	Total country	9608	15128	34467	59203	537035	562263	1111374	2210672
	R. Tarapacá	620	1003	2463	4086	11631	11773	20594	43998
	R. O’Higgins	175	275	655	1105	28.482	31.225	59.231	118.938
	R. Metropolitan	6116	9138	20426	35680	213479	214973	429298	857750
Residence	Urban area								
area	Total country	18485	29237	67011	114733	972976	1012758	2003042	3988776
	R. Tarapacá	1146	1990	4783	7919	23212	23339	40946	87497
	R. O’Higgins	324	503	1170	1997	44739	49100	92974	186813
	R. Metropolitan	12177	18243	40798	71218	418554	421530	836309	1676393
	Rural area								
	Total country	888	1391	3296	5575	121840	136092	267704	525636
	R. Tarapacá	86	67	237	390	723	743	1353	2819
	R. O’Higgins	44	63	200	307	13267	14609	28563	56439
	R. Metropolitan	168	308	763	1239	16673	18462	36539	71674

Among international migrants, of the total number of children and adolescents, 16.1% were between 0 and 4 years old, 25.5% between 5 and 9 years old and 58.4% between 10 and 19 years old. Of the total number of children and adolescents, 50.8% were male and 49.2% female. Of the total number of children and adolescents, 95.4 per cent lived in urban areas and 4.6 per cent in rural areas. In local areas, 24.3 per cent of the total number of children and adolescents were between 0 and 4 years old, 25.4 per cent between 5 and 9 years old and 50.3 per cent between 10 and 19 years old. Of the total number of children and adolescents, 51.0% were male and 49.0% were female. Of the total number of children and adolescents, 88.4 per cent lived in urban areas and 11.6 per cent in rural areas

Regarding school attendance, the census indicated that the percentage of the adolescent migrant population that had never attended school (0.6% vs. 0.1% in local adolescents) or that did not attend educational establishments at the time of the survey (20.5% vs. 8.8% in local adolescents) was always higher compared to the population of national adolescents. Similarly, with respect to child labour, it emerged that, at the national level, 21.9% of migrant adolescents were working for payment in cash or in kind as opposed to 10.3% of local adolescents, and that only 57% were studying as opposed to 77.8% of their local counterparts.

Regarding housing, 10.3% of adolescent migrants lived in materially precarious housing such as ‘*mediagua’* (improvised shelter), rented rooms or shared residences, compared with 2.5% of Chilean adolescents living in these conditions. Likewise, 0.3% of adolescent migrants were living in ‘*operativos de personas en tránsito*’, a type of shelter for unhoused transients. (Table 4 Census 2017 results by school attendance, child labour and housing).

**Table 4:** Census 2017 results by school attendance, child labour and housing

		Total country		*R*. Tarapacá	*R*. O’Higgins	*R*. Metropolitan
		N	%	N	N	N
School Attendance	International Migrants					
	Attends	95,536	80.11%	6472	1832	57,327
	Doesn’t attend currently	16,170	13.56%	1271	339	9839
	Has never attend	7550	6.33%	512	107	4673
	Non migrants					
	Attends	3,753,571	84.04%	75,174	200,592	1,444,993
	Doesn’t attend currently	242,370	5.43%	4766	13,601	93,691
	Has never attend	470,335	10.53%	9573	26,174	188,996
Child Labour	International Migrants					
	For a payment in cash or in kind	8083	24.71%	541	185	5089
	No payment for a relative	440	1.34%	57	7	215
	Was looking for a job	1699	5.19%	121	32	1039
	He/She was studing	21,020	64.25%	1337	387	12,470
	Performed household chores	1476	4.51%	165	26	810
	Other			373	95	2260
	Non migrants					
	For a payment in cash or in kind	114,574	11.04%	2384	5934	47,076
	No payment for a relative	6176	0.60%	147	246	2333
	Was looking for a job	29,222	2.82%	539	1580	11,458
	He/She was studing	863,985	83.26%	14,849	43,309	337,333
	Performed household chores	23,728	2.29%	412	1398	7564
	Other	72,130	6.95%	1558	4077	27,224
Housing	International Migrants					
	House	72,296	60.09%	5398	1789	37,413
	Apartment	34,704	28.85%	926	391	28,516
	Room	6266	5.21%	938	24	606
	Collective residence	1638	1.36%	119	59	4633
	Improvised shelter	3992	3.32%	752	24	663
	*In transit*	242	0.20%	21	0	88
	Homeless	15	0.01%	2	0	5
	Other	1155	0.96%	153	17	533
	Non migrants					
	House	3,862,392	85.56%	67,631	221,798	1,365,786
	Apartment	536,451	11.88%	17,380	15,779	343,140
	Room	18,330	0.41%	1240	472	11,455
	Collective residence	35,531	0.79%	1007	900	6986
	Improvised shelter	49,067	1.09%	2422	3939	15,941
	*In transit*	1111	0.02%	8	0	646
	Homeless	128	0.00%	3	0	57
	Other	11,402	0.25%	625	364	4056

*Child labor is only measured among migrant adolescents aged 15 to 19

**Fig. 1:**
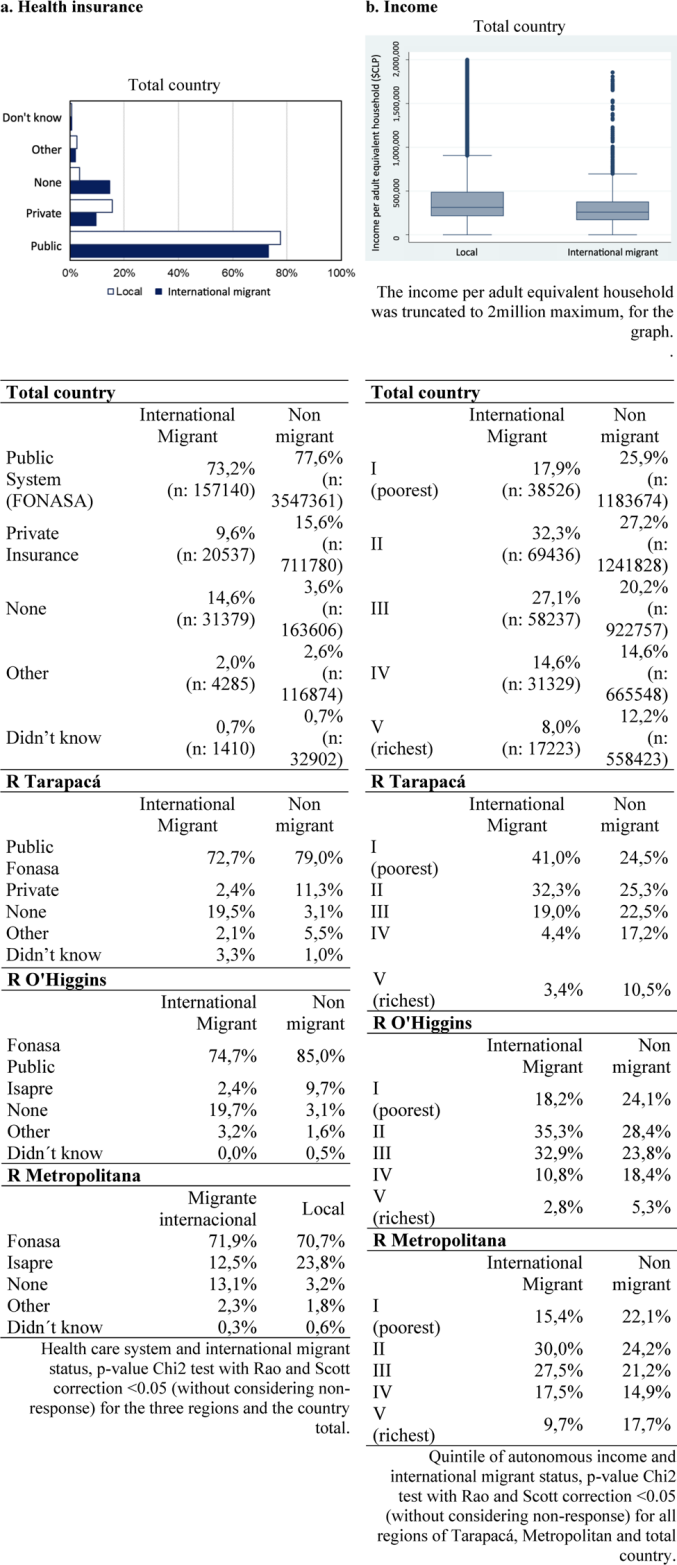
Results of CASEN 2020 socio-economic level and health insurance of international migrants and non migrants (locals) in Chile, adolescent populations

Concerning poverty, the CASEN survey found that, by 2020, there were approximately twice as many migrant children living in extreme poverty compared with Chilean children (11.0% vs. 5.6%) and that 30.9% of households with migrant children experienced medium to critical levels of overcrowding vs. 11.2% of Chilean households with children. The percentage of children and adolescents who reported not having any type of health insurance was higher among migrants than among locals, up to six times in some regions of the country. Finally, there was a statistically significant association between being a migrant children and lacking health insurance at the national level: 15.3% vs. 4.3% of local children and adolescents (Fig. 1 Results of CASEN 2020 socio-economic level and health insurance).

Furthermore, social vulnerability is confirmed by the qualitative data. A social worker, for example, confirmed that the educational system does not have enough availability for all migrant children and adolescents:

“We are unable to cope with everything related to schools and healthcare. Schools are truly saturated, and migrant children continue to arrive, leaving them without receiving education” (Social worker, women).

A migrant woman mentioned difficulties to access to a house for herself and her children, giving details about her living arrangements at the time:

“I haven’t been able to find a house. I spent all I have to rent this room (…), the money just disappeared. So, I live in a room, but it’s difficult to share the bathroom with unknown neighbours, it’s very difficult, especially for my daughter who is a teen now (…). Upstairs it’s horribly hot, because it’s like that, the sun shines through the whole window and in the summer, we can’t sleep, the children can’t stay there” (women, Bolivian).

### II) Epidemiological profile and healthcare needs

Regarding the reported causes of hospital discharges in adolescents, strikingly, the main cause of hospital discharges in migrant adolescents was pregnancy, childbirth or puerperium, accounting for 22.75% of discharges compared to 4.42% for their Chilean peers in 2021. This was followed by traumatic injuries, poisoning and other external causes (18.75% in migrant children and adolescents vs. 13.21% in locals), and diseases of the digestive system (8,67% in migrant children and adolescents vs. 14,6% in locals) or diseases of the respiratory system (2,4% in migrant children and adolescents vs. 6,08% in locals. Another interesting finding was that hospital admissions among migrant children and adolescents occurred in greater proportion in the adolescent age group (61%), while in Chilean children and adolescents, these were concentrated in the childhood age group (40.2%). Taking into account gender, female migrant adolescents accounted for 56.9% of EHs compared to 46.6% of Chilean adolescents. This disproportion was related to the higher prevalence of pregnancy, childbirth or puerperium among young women in the context of migration. When analysing the second cause of hospital discharge of migrant children and adolescents, traumatic injuries, poisoning and other external causes, it was found that a specific cause highly related to housing conditions and overcrowding, such as ‘burns and corrosions’, was more present among migrant adolescents (13.8%) than in non-migrants (9.3%) in the year 2021. Table 5 Hospital Discharges in children and adolescents 2021 according to discharge diagnoses.

**Table 5:** Hospital Discharges in children and adolescents 2021 according to discharge diagnoses

	**International migrants**			**Non migrants**		
	0 a 9 years old	10 a 19 years old	Total (0-19)	0 a 9 years old	10 a 19 years old	Total 0-19
**Total**	**1113**	**1740**	**2853**	**108126**	**72706**	**180832**
Main Diagnosis (CIE-10)						
C1 Infectious and parasitic diseases	70	19	89	5264	1260	6524
C2 Tumours (Neoplasms)	64	63	127	3633	3433	7066
C3 Blood diseases	27	18	45	1393	768	2161
C4 Endocrine and nutritional diseases	20	20	40	1496	2154	3650
C5 Mental and behavioural disorders and neurological development	3	99	102	348	7507	7855
C6 Nervous system diseases	57	40	97	3415	1975	5390
C7 Diseases of the eye and its appendages	1	4	5	479	249	728
C8 Diseases of the ear and mastoid process	4	2	6	351	403	754
C9 Diseases of the circulatory system	7	22	29	676	1050	1726
C10 Diseases of the respiratory system	152	44	196	14085	4157	18242
C11 Diseases of the digestive system	100	234	334	9146	13039	22185
C12 Diseases of the skin and subcutaneous tissue	21	24	45	1396	1574	2970
C13 Diseases of the musculoskeletal system and cognitive tissue	16	32	48	1255	3599	4854
C14 Diseases of the genitourinary system	52	67	119	10138	5598	15736
C15 Pregnancy, childbirth and puerperium	0	649	649	0	7848	7848
C16 Certain conditions originating in the perinatal period	112	0	112	26619	0	26619
C17 Congenital malformation, deformity and chromosomal anomalies	63	42	105	8853	2139	10992
C18 Not classified	42	32	74	3271	1532	4803
C19 Traumatic injuries, poisoning or other external causes	258	277	535	11902	11547	23449
C21 Specific proposals	44	52	96	4406	2874	7280
Secondary Diagnosis (External causes)						
Head trauma			71			3666
Elbow and forearm trauma			66			3447
Wrist and hand trauma			68			2212
Knee and leg trauma			45			2013
Other trauma			68			3362
Burns and corrosions			74			2203
Multiple injuries			33			1088
Foreign bodies in any part of the body			23			844
Intoxication by drugs or non-medical substances			56			3273
Frostbite			0			4
Unclassified traumatic, surgical or post-traumatic complications			31			1337

**Fig. 2:**
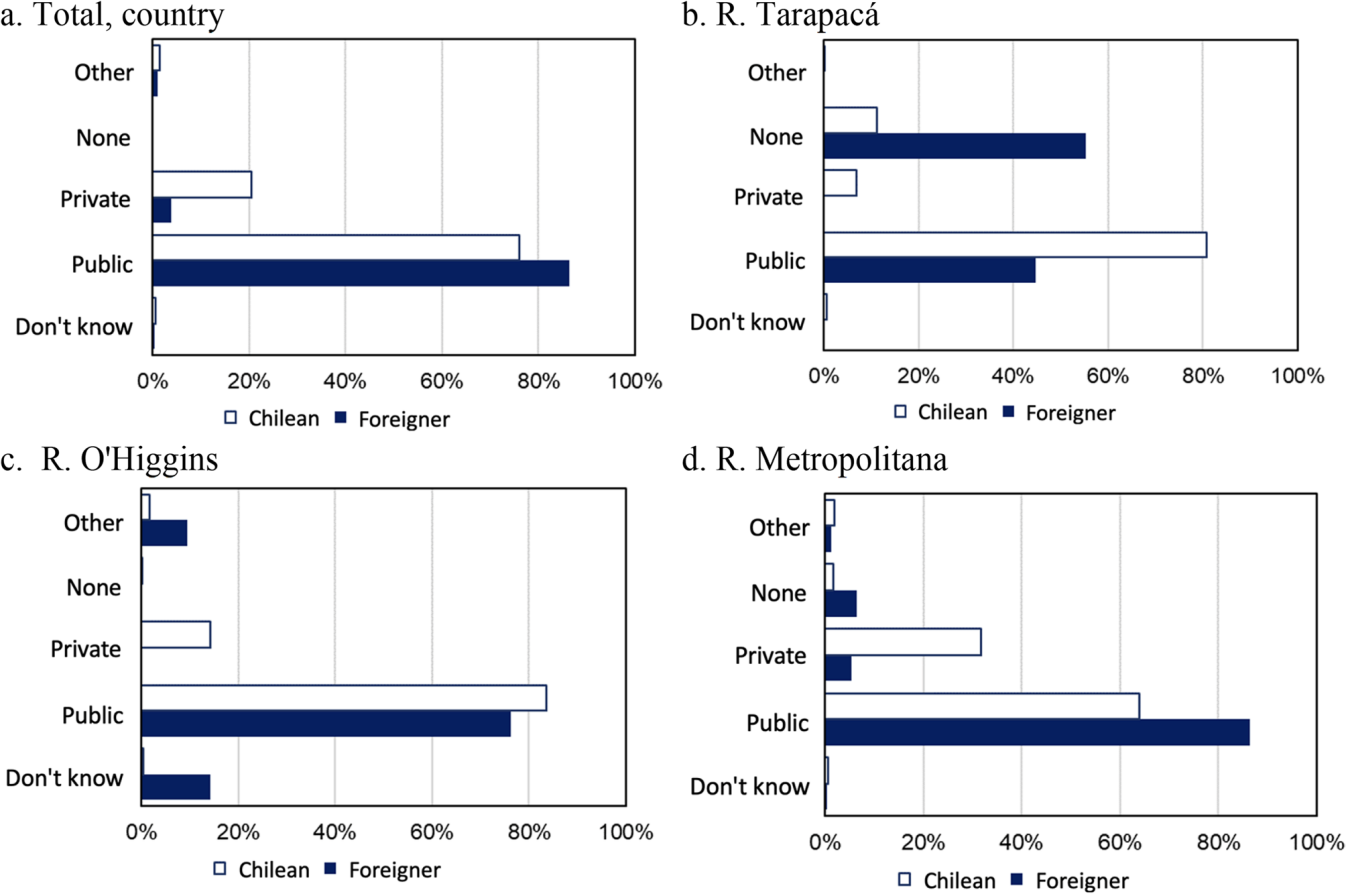
Hospital admissions of children and adolescents 2021 according to health insurance

Consistent with the results of the CASEN survey, among migrants, a higher proportion of people without insurance were represented in the EHs compared with their local peers. Of the total number of hospital admissions among children under 19 years old, there was a higher percentage of hospital admissions of children and adolescents without health insurance among international migrants than locals at the national level in 2021 (8.6% in migrants vs. 1.2% in locals). Figure 2 Hospital admissions of children and adolescents 2021 according to health insurance.

Finally, when analysing the Monthly Statistical Records of Public Primary Care (REM), we found that by 2021, *n* = 9,260 adolescents were attending check-ups under the adolescent health programme. However, the data showed issues of adolescent labour and lack of access to education, considering that, although in both populations the majority was studying (94.6% of local adolescents and 90.7% of migrant adolescents), school dropout rates were higher among migrants than among Chileans (3.7% v/s 1.2%) and 5.6% of migrant adolescents reported working, including under the worst forms of child labour or performing unpaid domestic service (5.6% v/s 4.2% of Chileans).

The qualitative data confirmed some of the characteristics regarding epidemiological profiles and health needs. On the one hand teen pregnancy was identified by healthcare professionals as a phenomenon that affects migrant adolescents:

“We also see now many teenage mothers, teenage migrant mothers and it is not the first child, they can be 3 or 2, 16 years old, 15 years old. The issue of adolescents, (parents) don’t bring them, they don’t think ‘my child is already an adolescent, I’m going to take him or her to see about reproductive health…’ no, nothing like that.”. (Nurse, women)

“There have been some cases of adolescents between 15 and 18 years old who have arrived pregnant. Often, they arrive with a partner, not with their parents, so we have to raise the issue of a violation of rights because they are still minors and are pregnant, their partner is an adult and sometimes with a big age difference, in these cases we take protective action for the girl and her baby.” (Psychologist, men).

Additionally, overcrowding and housing conditions were related to accidents, traumatic injuries etc., according to both migrants and healthcare teams:

“Many children are left alone. Their parents work, and the children take care of each other, or they are looked after by the neighbour across the street, a friend, or an uncle, it can be very unsafe” (Social worker, women).

“Here, the power goes out all the time, there are fires, accidents, because the houses are like this (made with waste material)” (men, Venezuelan).

Lastly, some interviewees reported incidents of school violence that resulted in hospital visits for minor injuries, revealing situations of discrimination and stigma experienced by migrant children and youth in school settings:

“At school, with all this bullying and everything, and also because of his behavior… there was an incident at school where they hit him. A boy pushed him and hit his head against the bathroom wall and also bent his thumb. So I had to take him to the hospital…” (woman, Venezuelan).

Unlike what epidemiological records indicate, the qualitative data did not delve into other specific health conditions that could affect migrant adolescents, despite the fact that they were included in the interview guidelines.

### III) Barriers to access primary healthcare programmes dedicated to adolescent health from the perspective of adult migrants and healthcare teams

The previous section identified barriers to access primary healthcare affecting migrant children and adolescents based on quantitative data, especially lack of insurance. This section presents results regarding these barriers based on the qualitative data of the study.

The first dimension identified is that preventive health services for the adolescent population were little known by adult migrants, even among those in charge of adolescents between 10 and 19 years old. This woman, for example, explained the situation of her 14-year-old sister:

“My sister has the device, the IUD, and she wants to have it removed because she does not feel well, the hormones and things like that, (…). That was in Ecuador, they had her it put in because she had a boyfriend and then she was with that man, otherwise she could have gotten pregnant… and now I want her to have it removed because it’s driving her crazy and she no longer has a boyfriend (…) but I don’t know where to take her, I don’t know where she can go, it will have to be a private gynecologist that you have to pay for”. (women, Venezuelan)

Another testimony showed that migrants were unaware of the healthcare programme dedicated to adolescents and the idea that prevention was unnecessary in the absence of illness, citing issues such as the incompatibility of multiple appointments with work obligations and the need to prioritise work:

“Yes, she has always been a very healthy child. Now she is developing, but we haven’t taken her to any check-ups, because she doesn’t get sick (…) besides, it is often difficult to take her, you have to ask for permission at work and they won’t give it”. (women, Colombian)

In fact, when we asked specifically about the adolescent health program, most interviewees were unaware of its existence, even though they had children of adolescent age:

“No, I didn’t know there’s care for adolescents. I only take the little ones, but the older one… no, nothing.” (woman, Bolivian).

Furthermore, school was identified as the place where adolescents received information on sexual and reproductive health, instead of primary healthcare services:

“I’ve never had to take them to a doctor’s office because at school they are informed all the time.” (women, Venezuelan).

This reported lack of knowledge was aligned with the perception of health professionals who argued that adolescent health in general is a topic that was poorly covered and that the strategies developed with schools are important, although they were not able to sustain them over time:

“In general it is very difficult to reach adolescents, they hardly come to check-ups. How do we keep them engaged? We have to go and do outreach activities in schools. And this is a problem that we identified some time ago, adolescents don’t come. So this year we are already creating an intervention plan to go to the high school. Because in general, regardless of the nationality, they don’t reach us.” (Psychologist, men).

Healthcare teams also recognised that seeking care on their own, without an adult, may be a barrier for adolescents, despite the fact that Chile recognises the right of people over 14 years old to access healthcare without the authorisation of a responsible adult (regulation A15/11, 2016):

“When adolescents come here to the clinic they must come with their father, mother, a responsible adult.

Researcher: - Even if they are over 14 years old?

-Yes, up to the age of 18. If they have already turned 18 they can come alone, but otherwise they must come with a responsible adult.”. […] (Nurse, men).

However, despite the lack of knowledge and barriers to adolescent healthcare, both healthcare teams and migrant adults recognised the need to have tools to address different types of healthcare, such as mental health:

“We understand that in Venezuela the issue of mental health, the psychologist, to say that you go to a psychologist is to say that you are crazy, that is something that is not discussed, something that is very stigmatised. And what we have been seeing now is that among adolescents, what parents are asking for is psychological support, issues of depression, suicide attempts, issues that we do not usually handle with migrant families but that are emerging.” (Social worker, women).

The needs identified for adolescents go beyond the traditional realm of sexual and reproductive health, and mental health and nutritional health issues have been increasingly recognised. For example, one nurse argued:

“I think that many mothers ask for psychological help for their adolescent children, which has been on the rise, and above all one sees it among Venezuelans (…) they say that the adolescent is grown up, but then you are not seeing him as an adolescent, and as he is grown up you expect him to be able to take on adult responsibilities, but that is not how it is, and we are not seeing it.” (Social worker, women).

The migrant adults interviewed also mentioned mental health as an issue, while also indicating that they lacked the tools to handle the changes experienced by their children, in terms of mental health and nutrition:

“My son, he has obviously been experiencing changes in his body, he feels strange because he has said that he feels strange (…). And those are the changes he’s had, all the changes that children go through. And obviously you’d like to have the support of someone who can see him, because obviously doctors and all that know a bit more about these things. There was a time when he was rebellious, yes, like when the change came that way, he was like rebellious, he also put on weight, he became chubby, so many changes.” (women, Colombian).

The qualitative data reinforce the findings of the quantitative component by giving greater depth to phenomena such as the lack of knowledge regarding healthcare services dedicated to adolescents, the characteristics of adolescent pregnancy and the daily living conditions of migrant adolescents in Chile.

## Discussion

This is one of the first studies to describe the health of adolescent migrants in the Southern Cone, based on quantitative and qualitative data. Although the data are descriptive and exploratory, they are of primary importance for the region, as they allow us to identify areas in which adolescent migrants are facing vulnerabilities and inequities that generate cumulative medium and long-term effects on their health [24]. While similar studies have been conducted with migrant children [25–27], few of them characterise the general aspects of the social and health status of adolescents in a context where they increasingly participate in migration flows [16, 25].

Firstly, the study shows that adolescent migrants in Chile are more likely than their local peers to be out of school, to be involved in various forms of work and to have no health insurance. From a social determinants of health perspective, education ‘is strongly associated with life expectancy, morbidity, health behaviours, and educational attainment and plays an important role in health by shaping opportunities, employment, and income’ [28]. Conversely, child labour has been shown to negatively impact physical health, including nutritional status, physical growth, work-related illnesses/symptoms, musculoskeletal pain, HIV infection, infectious diseases, tuberculosis and eyestrain [29]. It can also have consequences for mental health, especially if associated with school dropout and exposure to violence and abuse. Although this study does not show impacts, the results coincide in the risk that work involvement and school absenteeism pose to the health of this specific group.

Secondly, lack of social security is a frequent barrier for international migrants, especially those with an irregular migratory status [30]. The persistence of this barrier is not consistent with the current constitutional, legislative and regulatory provisions in Chile, which do not distinguish between locals and migrants, regardless of their legal status [31]. This shows that the norm is not being fully implemented by all sectors and that fear, lack of knowledge [32] and the exacerbation of a climate of racism [33] hinder people’s access to preventive services. Studies with migrants in the United States, Chile and Canada have shown that lack of health insurance among children and adolescents is associated with the overuse of emergency services and hospitalisation for preventable causes [34, 35]. In this area, it is worrying that healthcare teams themselves are creating barriers for adolescents by placing discretionary obstacles (such as the requiring an adult to be present when seeking care) in response to adolescents’ health needs [13].

Thirdly, another finding of the study is related to housing conditions and habitability, both of which are considered social determinants that negatively affect the migrant population in large Latin American cities [36]. The study shows that more than 10% of adolescent migrants surveyed in the 2017 Census were living in rented rooms, informal shelters, shared housing or were unhoused. Additionally, 30.9% of migrant families with children and adolescents experienced medium to critical overcrowding, according to the CASEN 2020 survey. Although it is not possible to establish causal relationships, hospital admissions due to ‘traumatic injuries, poisoning and other external causes’, which are the second leading cause for migrant adolescents, and specifically admissions related to burns and corrosions, may be linked to conditions of vulnerability and precarious housing, as well as participation in risky activities, mental health issues and self-harm [37]. Qualitative data also reveal exposure to accidents resulting from school violence, which may be linked to stigma, discrimination, and xenophobia. This needs further research and action, as it might represent unsafe, harming educational environments for migrant children in our country.

Fourthly, this study presents results regarding teenage pregnancy. This issue has been a top public health priority in Chile and has been addressed through targeted policies leading to a downward trend, among adolescents between 10 and 19 years old at the national level in the past decade [38]. However, studies warn of persisting social and territorial inequalities, as the poorest districts of the country and/or rural sectors still concentrate the highest numbers of pregnant adolescents [39]. In addition to this finding, the data reported here show that the phenomenon affects migrant adolescents up to five times more than their local peers. Furthermore, the conditions in which these pregnancies are experienced are highly vulnerable, as reported by healthcare teams, who refer to situations of multiple pregnancies, unaccompanied adolescents and/or significant age difference with the partner. All these factors increase the riskiness of these pregnancies and the negative consequences they can have on maternal and newborn health. This result is also consistent with other studies that show associations between migration, conditions of vulnerability, adolescence and early pregnancy, showing that the issue requires further studies from a regional perspective [19, 40].

Finally, the study also found lack of knowledge and other barriers to accessing primary care services aimed at the adolescent migrant population. While recognising that the lack of policy prioritisation affects all adolescents and that it is necessary to implement intersectoral initiatives such as the ones carried out in educational establishments [41], healthcare teams and migrant adults recognise that adolescent migrants have specific needs that are not being covered by primary health care services. So far, the adolescent health program has lacked an intercultural approach [5] and interactions between healthcare teams and migrant adolescents typically occur only when a specific need arises (e.g., accidents, pregnancy, illness). The preventive focus of this type of service requires greater involvement of healthcare personnel in social activities with the migrant community, as well as their presence in spaces frequented by adolescents, such as schools, recreational areas, and cultural centers [42, 43]. Coordination with intercultural leaders and facilitators has also proven beneficial in strengthening relationships with migrant communities in other contexts [44].

It is important to also highlight that the majority of identified social inequalities in health in this unique study are deeply rooted in systemic, persistent, historical inequities that need urgent attention [42]. Unequal opportunities to access to healthcare services and experiencing additional perceived barriers to care are the visible side of xenophobic, colonial stereotypes and structural discrimination processes that require urgent attention in Chile and the region. All form of stigma and discrimination represents not only a measure of social injustice but also a serious threat to population health [33]. This is especially significant during early stages of life when the sense of self-identify, self-value and purpose, as well as the quality and meaning of social interactions, are initiated and developed. Public policies in Chile need to address these urgent matters by guaranteeing effective access, monitoring quality of healthcare and shortening existing gaps in coverage between migrants and locals.

Barriers to involving adolescents themselves in the study and the lack of in-depth data on issues such as mental health, nutritional health, environmental health and other related topics are the main limitations of this study. Furthermore, quantitative and qualitative data were collected in different years. Moreover, the available quantitative data did not indicate the percentage of adolescent health program coverage among the migrant population, making it possible to identify barriers only through qualitative data. Some data were also collected in pandemic and post-pandemic contexts, so it is possible that some features of the context may have changed. The qualitative sample size is small and does not aim at being representative. Despite these limitations, this is the first study of this kind in the country and raises important issues related to social inequalities in health among migrant adolescents in Chile and also guides towards novel questions for future investigation. Future research is needed to further explore the issues raised in this study, as well as to investigate the resources available and collective strategies adolescents may have to address the needs we have identified.

## Conclusions

Adolescents are an important population group in Latin America and many of them migrate. This mixed methods study shows that adolescent migrants face inequities and vulnerabilities that prevent them from fully exercising their right to health, posing risks for their present and future. Although important efforts have been made to make the health needs of adolescents visible, priority actions are still needed for this specific group in order to guarantee their basic rights such as access to education, housing and health, increase the acceptability and coverage of preventive care, as well as strengthen their participation in the social and health decisions that affect them.

## Data Availability

The datasets generated and/or analysed during the current study are available in the Universidad del Desarrollo repository, [https://repositorio.udd.cl/items/76ff71cf-4de7-4733-bbba-e1dd8520138a](https:/repositorio.udd.cl/items/76ff71cf-4de7-4733-bbba-e1dd8520138a).
